# COVID-19: The Immune Responses and Clinical Therapy Candidates

**DOI:** 10.3390/ijms21155559

**Published:** 2020-08-03

**Authors:** Sareh Zhand, Marie Saghaeian Jazi, Saeed Mohammadi, Roozbeh Tarighati Rasekhi, Ghassem Rostamian, Mohammad Reza Kalani, Aida Rostamian, Jacob George, Mark W Douglas

**Affiliations:** 1 School of Biomedical Engineering, University of Technology Sydney, Sydney, NSW 2007, Australia; sareh.zhand@uts.edu.au; 2 Department of Microbiology, Faculty of Biological Sciences and technology, Shahid Beheshti University, Tehran 1983969411, Iran; 3 Metabolic Disorders Research Center, Golestan University of Medcial Sciences, Gorgan 4934174515, Iran; marie.saghaeian@goums.ac.ir; 4 Stem Cell Research Center, Golestan University of Medical Sciences, Gorgan 4934174515, Iran; s.mohammadi@goums.ac.ir; 5 Infectious Diseases Research Center, Golestan University of Medical Sciences, Gorgan 4934174515, Iran; 6 Department of Radiology and Imaging Sciences, School of Medicine, Emory University, Atlanta, GA 30322, USA; roozbeh.rasekhi@emory.edu; 7 Reliance GP Super-Clinic, Gosford, NSW 2250, Australia; ghassem.rostamian@reliancehealth.com.au; 8 Medical Cellular and Molecular Research Centre, Golestan University of Medical Sciences, Gorgan 4934174515, Iran; kalanimr@yahoo.com; 9 Department of Clinical Sciences, Faculty of Veterinary Science, Islamic Azad University of Karaj, Alborz 3149968111, Iran; aida.rostamian@yahoo.com; 10 Storr Liver Centre, Westmead Institute for Medical Research, Westmead Hospital and University of Sydney, Sydney, NSW 2145, Australia; 11 Centre for Infectious Diseases and Microbiology, Marie Bashir Institute for Infectious Diseases and Biosecurity, University of Sydney at Westmead Hospital, Sydney, NSW 2145, Australia

**Keywords:** SARS-CoV-2, COVID-19, MERS-CoV, SARS-CoV, clinical trial, anti-viral, anti-parasite, molecular immune response, immunotherapy, adjunctive therapy

## Abstract

The pandemic of coronavirus disease 2019 (COVID-19), with rising numbers of patients worldwide, presents an urgent need for effective treatments. To date, there are no therapies or vaccines that are proven to be effective against severe acute respiratory syndrome coronavirus 2 (SARS-CoV-2). Several potential candidates or repurposed drugs are under investigation, including drugs that inhibit SARS-CoV-2 replication and block infection. The most promising therapy to date is remdesivir, which is US Food and Drug Administration (FDA) approved for emergency use in adults and children hospitalized with severe suspected or laboratory-confirmed COVID-19. Herein we summarize the general features of SARS-CoV-2’s molecular and immune pathogenesis and discuss available pharmacological strategies, based on our present understanding of SARS-CoV and Middle East respiratory syndrome coronavirus (MERS-CoV) infections. Finally, we outline clinical trials currently in progress to investigate the efficacy of potential therapies for COVID-19.

## 1. Introduction

The outbreak of severe acute respiratory syndrome coronavirus 2 (SARS-CoV-2) infection has rapidly spread worldwide and produced unprecedented social and economic harm. Coronavirus disease-19 (COVID-19) is an illness caused by this novel coronavirus and can present with symptoms ranging from mild or minimal respiratory symptoms to acute respiratory distress syndrome (ARDS). During the last twenty years, coronaviruses have caused three epidemics of lethal diseases: severe acute respiratory syndrome (SARS), Middle East respiratory syndrome (MERS) and COVID-19. SARS-CoV-2 seems to be less virulent than SARS-CoV or MERS-CoV with mortality rates of 3.4%, 9.6% and ~35%, respectively [1] but appears to be more infectious.

This rapid spread of COVID-19 has generated intense research interest to find a cure or a vaccine. However, there are no Food and Drug Administration (FDA)-approved drugs for the treatment of COVID-19, although remdesivir, an investigational antiviral drug, is available through an FDA emergency use authorization [2]. Several medicines with antiviral or immunomodulatory mechanisms have been recommended as potential investigational therapies, many of which are currently being studied in clinical trials. To date, therapies with proven benefit in certain groups include remdesivir, dexamethasone and convalescent plasma [3,4,5]. This review describes the structure of SARS-CoV-2, its molecular and immune pathogenesis and summarizes current knowledge about the potential mechanisms for off-label agents widely used to treat COVID-19.

## 2. Virology of SARS-CoV-2

SARS-CoV-2 (about 120 nm in diameter) is an enveloped, positive-sense single-stranded RNA (26–32 kb) virus that belongs to the genus *Betacoronavirus* of the family *Coronaviridae* in the Nidovirales order. The subgroups of the family are alpha (α), beta (β), gamma (γ) and delta (δ) coronaviruses [6]. During the last two decades, several highly pathogenic human coronaviruses have emerged including SARS-CoV in 2002–2003, MERS-CoV in 2012 [1] and SARS-CoV-2 in 2019.

**Figure 1:**
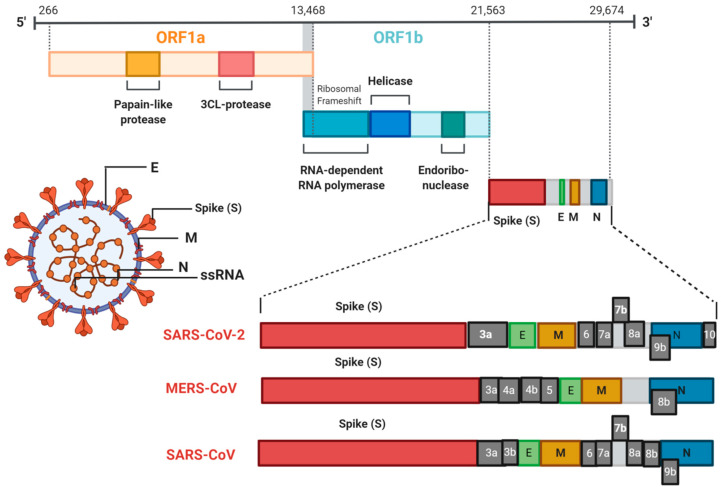
Coronavirus genomic organization. Coronaviruses are enveloped particles 100–160 nm in diameter with a 26–32 kb single stranded RNA (ssRNA) genome. Severe acute respiratory syndrome coronavirus (SARS-CoV), Middle East respiratory syndrome coronavirus (MERS-CoV) and severe acute respiratory syndrome coronavirus 2 (SARS-CoV-2) share a common open reading frame 1 (ORF1)a/b which encodes a polyprotein. The other ORFs are responsible for coding the four main structural proteins: spike (S), envelope (E), membrane (M) and nucleocapsid (N) proteins plus several accessory proteins.

Several phylogenetic analyses of the origin of the SARS-CoV-2 suggests bats are the most likely animal reservoir. Full-genome evolutionary analysis suggests that SARS-CoV-2 is closely related to BatCoV RaTG13 with 96.3% sequence similarity [7] and two SARS-like coronaviruses derived from bats (bat-SL-CoVZC45 and bat-SL-CoVZXC21) with 88% similarity, compared to only 79% and 50% similarity to SARS-CoV and MERS-CoV, respectively [8] (Figure 1). Transmission of SARS-CoV-2 from bats to humans is likely to have involved an intermediate host, as seen for SARS-CoV (palm civets) and MERS-CoV (dromedary camels). Metagenomic sequencing suggests that Pangolins could be the intermediate hosts due to the genome similarity of Pangolin-associated coronavirus to SARS-CoV-2 (approximately 85.5–92.4%) [9]. A comprehensive synonymous codon usage (RSCU) bias analysis suggests SARS-CoV-2 might be a recombinant between bat coronavirus and an unknown coronavirus with the same codon usage bias as snake coronavirus [10]. Population genetic analysis of 10^3^ SARS-CoV-2 genomes by Tang et al. [11] indicated that this virus has evolved into two main types, L and S types. The L type (~70%) is more prevalent than the S type (~30%), due to its higher transmission and/or replication rates, although the S type is older in evolutionary terms [11].

## 3. Coronavirus Entry and Replication

All coronaviruses have specific genes downstream of the open reading frame 1 (ORF1) that encode for proteins necessary for viral replication and generation of the nucleocapsid and spike proteins [12]. SARS-CoV-2 has 14 ORFs encoding for 27 proteins. ORF1a/b and ORF1a are located at the 5′ end of the SARS-CoV-2 genome and code for the pp1ab and pp1a polyproteins, respectively. These polyproteins contain 15 non-structural proteins (NSP) including NSP1 to NSP10 and NSP 12 to NSP 16. The 3′ end of the genome encodes four structural proteins including spike glycoprotein (S), envelope protein (E), matrix protein (M), nucleocapsid protein (N) and eight accessory proteins (3a, 3b, p6, 7a, 7b, 8b, 9b, and ORF14). Despite similarity in the overall amino acid composition of SARS-CoV-2 and SARS-CoV, remarkable amino acid differences have been identified [13].

The glycoprotein spikes located on the outer surface of coronaviruses work as multifunctional proteins contributing to host receptor binding, cellular tropism and pathogenesis [14]. The amino acid sequence of the SARS-CoV-2 S-protein is 76.47%, similar to that of SARS-CoV, leading to the same structural and electrostatic characteristics at the interaction interface [15]. The S-protein of SARS-CoV-2 resembles a mixture of bat SARS-CoV and an anonymous Beta-CoV [16], with the 3-D structure of the receptor binding domain (RBD) region maintaining similar van der Waals interactions [17].

Both SARS-CoV and SARS-CoV-2 use angiotensin converting enzyme II (ACE2) to enter human and bat cells [18]. The interaction between glutamine 493 in the RBD region of the SARS-CoV-2 spike protein and lysine 31 on the human ACE2 receptor plays an important role in viral binding and entry [19]. Cryo-electron microscopy (Cryo-EM) structure analysis of the spikes shows that the binding efficiency of the SARS-CoV-2 S protein to ACE2 is 10 to 20-fold higher than for SARS-CoV [20]. A single N501T mutation in the spike protein of SARS-CoV-2 significantly enhances the binding affinity for its receptor, contributing to the high transmissibility of COVID-19 [19].

The spike protein of coronaviruses forms a trimer that generates a crown-like structure on the envelope, giving this virus family its name (corona = crown) [14]. This large glycoprotein is divided functionally into the S1 and S2 domains. The S1 domain (amino acids 15–680 in SARS-CoV) is responsible for virus binding to its receptor on the target cell and comprises two sub-domains: the N-terminal and the C-terminal of RBD which consists of 193 amino acids (318–510 in SARS-CoV). The S2 domain of the spike protein (amino acids 681–1255 in SARS-CoV) participates in cell membrane fusion [17,21]. Cellular proteases including human airway trypsin-like protease (HAT), transmembrane protease serine 2 (TMPRSS2) and cathepsin trigger cleavage of the S protein trimer in SARS-CoV, while furin makes the MERS-CoV S protein competent for fusion. TMPRSS2 is a serine protease localized to the plasma membrane that is responsible for proteolysis of the S protein after receptor-binding [22]. However, the mechanism of enhanced membrane invagination seen during SARS-CoV-2 endocytosis has not been identified [23].

The initial step during SARS-CoV-2 infection is the binding of S to its host receptors, inducing uptake of virus particles by endocytosis and fusion between host and viral membranes [18]. Following membrane fusion and viral entrance, viral genomic RNA is released into the cytoplasm of infected cells. This uncoated RNA acts as a template for translation of the two polyproteins, pp1a and pp1ab. The nsp3 and nsp5 encodes two protease enzymes named papain-like proteases (PLpro) and chymotrypsin-like protease (3CLpro) which cleavage between pp1a and pp1ab to form nsps1–11 and 1–16, respectively [24]. A large number of non-structural proteins make the replication–transcription complex (RTC) in double-membrane vesicles. The accessory and structural proteins are generated by translation of a nested set of subgenomic RNAs synthesized by the RTC. Moreover, a negative strand RNA template is transcribed and forms a complex with intermediate products for transcription of positive-sense mRNAs including the progeny genome and subgenomic RNA, mainly mediated by RNA-dependent RNA polymerases (RdRp) [25]. In the infected cell, newly synthesized viral genomic RNA, the structural nucleocapsid and envelope glycoproteins are assembled into viral particles in the endoplasmic reticulum (ER) and Golgi apparatus. In the final step, budding of the viral particles occur through fusion of the plasma membrane and virion-containing vesicles, leading to release of newly formed virions [23].

## 4. Pathogenesis of COVID-19

Although the immunopathogenesis of COVID-19 is not well understood, its clinical symptoms and signs vary from fever and fatigue to severe respiratory complications and multi-organ failure [26]. Understanding the immunopathogenesis of COVID-19 and the differences between affected individuals is essential for designing therapies and decreasing mortality. One approach is to study similar viruses such as SARS-CoV and MERS-CoV and learn from previously described host–pathogen interactions [27].

As discussed, SARS-CoV-2 employs ACE2 as the host cell receptor. ACE2 is highly expressed on many different cell types in a variety of human organs, including lung alveolar epithelial cells and small intestinal epithelial cells, both of which are considered potential SARS-CoV-2 routes of transmission. Overall, ACE2 is expressed widely in the main target organs of SARS-CoV-2, as well as organs that play less important or even unknown roles in COVID-19 pathophysiology [28]. It has been reported that SARS-CoV also infects immune cells such as T lymphocytes, monocytes and macrophages [29], but it remains poorly defined as to what extent SARS-CoV-2 also targets these cells. A number of studies are exploring the immune response to the SARS-CoV-2 [30], with most suggesting that the immune response is disturbed due to aberrant activation of monocytes/macrophages [31], elevation of pro-inflammatory cytokines [32], depletion of lymphocytes [33] and deployment of large numbers of neutrophils [34], but the complex mechanisms involved are still not well understood.

## 5. The Innate Immune Response to SARS-CoV-2 and Virus-Induced Degradation of RNA Sensors

Similarly to other viral infections, the expression of type I interferons (TI IFN), such as interferon (IFN)-α and IFN-β, determine the innate immune response to SARS-CoV-2 infection [35]. Pathogen associated molecular patterns (PAMPs) such as viral RNA are sensed by pattern recognition receptors (PRRs) on innate immune cells including antigen presenting cells (APCs), resulting in their activation [36]. Endosomal PRRs including Toll-like receptors (TLR-) 3, 7/8 and 9 are responsible for detection of SARS-CoV-2 [24]. These TLRs are mainly expressed in macrophages and dendritic cells, which are involved in innate immunity [37]. Activation of the TLRs leads to nuclear translocation of NFκB and IFN regulatory factor 3 (IRF3) transcription factors resulting in TI IFN overexpression and the release of pro-inflammatory cytokines (interleukin (IL)-1, IL-6 and Tumor necrosis factor TNF-α), which have been reported to be overexpressed significantly in SARS-CoV-2 infection in association with “cytokine storm” [37,38]. TI IFNs and these other cytokines also amplify their own expression [39], priming the adaptive immune response or pathogen clearance. However, the inflammatory status can be excessive and is one of the major determinants of COVID-19 disease [27].

In addition to TLRs, several other receptors including RIG-I-like receptors (RLRs) [27], C-type lectin-like receptors (CLRs) [40], NOD-like receptor (NLR) especially NLRP3 inflammasome [41], and free molecule receptors such as STING, cGAS, IFI16 and DAI can be involved in the recognition of viruses and in mediating immune response-related signaling pathways [42]. RLRs are comprised of the H family members RIG-I (DDX58), MDA5 (IFIH), and LGP2 which recognize the genomic structure of RNA viruses such as SARS-CoV-2 [27]. NLRs are a subclass of PRRs which are comprised of a conserved NOD structure and classified into three main categories according to their functions: inflammasome (NLRP1, NLRP3, NLRP6, NLRC4, NLRC5W, and AY2); embryo regenerative and regulatory NLRs [25,41]. CLRs are soluble PRRs mostly expressed in myeloid cells responsible for phagocytosis, maturation of dendritic cells (DCs) and chemotaxis and have been proposed to be associated in SARS-CoV-2 immunopathogenesis [40,43].

Like other viral infections, the SARS-CoV-2 antigens are processed by specific antigen presenting cells (APCs) including macrophages and dendritic cells [44]. The antigens are then presented by major histocompatibility complexes (MHC) or human leukocyte antigens (HLA) to specific cytotoxic T lymphocytes (CTLs) [45]. It is thought that SARS-CoV-2 relies on MHC I, like SARS-CoV and MERS-CoV, with minimal contribution by MHC II [46]. HLA polymorphisms have been correlated with an altered risk of SARS-CoV and MERS-CoV [47], so it is predicted that polymorphisms in HLA-A, HLA-B, HLA-DR B1, HLA-Cw and HLA-DQB1 could also exert an effect on the immunopathogenesis of SARS-CoV-2 [47].

However, like SARS-CoV and MERS-CoV, SARS-CoV-2 is capable of escaping the immune response [48]. For example, SARS-CoV can successfully shift ubiquitination and enhance degradation of RIG-I/MDA5 RNA sensors, TLR3, TLR7 and TLR8 [49]. Furthermore, SARS-CoV and possibly SARS-CoV-2 can suppress innate immune responses without triggering the anti-viral response machinery, by counteracting NFκB and TI IFN signaling [50]. Moreover, protective immunity against SARS-CoV-2 has not been reported and the virus is capable of immune escape and proliferation in infected organs [36].

## 6. Regulation of Adaptive Immune Response in SARS-CoV-2 Infection

### 6.1. T-Cell Mediated Immune Responses Against SARS-CoV-2 and Mechanisms of Immune Escape

T lymphocytes play a crucial role in the anti-viral immune response to coronaviruses [32]. Cluster of differentiation CD4+ and CD8+ T cells maintain the balance between the immune response against these pathogens and immune tolerance [51,52]. Antibody-producing B cells are activated by CD4+ T cells, while CD8+ T cells are cytotoxic to virus-infected cells [53]. During SARS-CoV infection, the majority (approximately 80%) of inflammatory cells infiltrating the lungs of affected individuals are CD8+ T cells [54] and T cells can survive in infected tissues [55]. In contrast, CD4+ T cells are more susceptible than CD8+ cells to infection by MERS-CoV and SARS-CoV [56]. During SARS-CoV-2 infection, a reduction in the number and quality of CD4+ T cells could be associated with decreased activation of B lymphocytes, with reduced levels of virus-specific neutralizing antibodies and pro-inflammatory cytokines (including IL-1β, IL-6 and TNF-α), leading to defective clearance of virus from infected organs [56]. Cooperating with innate immune cells, helper T cells are involved in producing major pro-inflammatory cytokines via NF-kB activation, which is responsible for initiating the production of pro-inflammatory cytokines involved in the cytokine storm [24]. IL-17 produced by Th17 cells mediates the activation of monocytes/macrophages, dendritic cells (DCs) and neutrophils and enhances the production of cytokines (IL-1, LL-6, IL-8, IL-21, TNF-α, and Monocyte chemoattractant protein-1 (MCP-1)) from these cells [30]. The possible role of IL-17 and related altered cytokines are highlighted in SARS-CoV-2 infection [57].

One of the important methods that SARS-CoV-2 may use to evade T cell-mediated immune responses is the induction of apoptosis, similar to previous reports in MERS-CoV and SARS-CoV, by enhancing the binding of T-cells to B-cell lymphoma-extra-large (Bcl-xL) molecules [58]. Although this might result in T-cell depletion, recent findings suggest that the T cell response to structural proteins of coronaviruses (including the S, M and N proteins) persists, providing effective and long-term T-cell responses against COVID-19, which conveys promise for developing vaccines [58].

### 6.2. Humoral Immune and Antibody Responses to Coronavirus Infection

All subtypes of B lymphocytes are involved in the humoral immune response against coronavirus infections [59]. Several antibodies which have been isolated and designated from MERS-CoV and SARS-CoV infected patients are MERS-GD27, CDC-A2, CDC-C2, MCA1, CSC-C5, CDC-A10, and MERS-GD33. We could expect that structurally similar antibodies would be isolated from SARS-CoV-2 patients [60]. The RBD of the SARS-CoV-2 S protein has been identified as an immunodominant and highly specific target for antibodies in affected patients [61]. Since the RBD of the S protein is poorly conserved among SARS-CoVs and other coronaviruses it is a promising antigen for detecting coronavirus specific antibodies, as demonstrated by Premkumar et al. using a large panel of human sera [62,63]. Zost et al. demonstrated that several human monoclonal antibodies (mAbs) targeting the S glycoprotein, including COV-2196 and COV-2130, showed potential neutralizing activity and fully blocked the RBD from interacting with ACE2 [64]. Similarly, epitope mapping by Liu et al. identified a short list of 19 antibodies, equally divided between those targeting the RBD or N-terminal domains, confirming that both are immunogenic [65].

The complement system is an innate immune compartment able to recognize and destroy pathogens that works tightly with the humoral arm of immunity [66]. Viral proteins can mimic host proteins that regulate the complement system, helping the virus to evade immune recognition [67]. C3a and C5a are responsible for the inflammatory response activation of innate immune cells. Thus, inhibiting C3a and C5a during infection with MERS-CoV, SARS-CoV or SARS-CoV-2 could be introduced as promising targets to treat severe lung disease [68].

### 6.3. Cytokine Responses Across COVID-19; the Significance of IL-6 in Cytokine Release Syndrome (CRS)

Although several management strategies are evolving for COVID-19, severe disease results in the ARDS associated with a cytokine release syndrome (CRS). CRS is caused by vast numbers of immune cells producing pro-inflammatory cytokines in a positive feedback loop [69] and is one of the key elements contributing to the mortality and morbidity of COVID-19 [70].

CRS is not specific to SARS-CoV-2 infection and has been demonstrated for MERS-CoV and SARS-CoV, as well as for influenza A subtypes H5N1 and H7N9 [71]. The hallmarks of CRS are elevated serum concentrations of pro-inflammatory cytokines including interleukin-6 (IL-6) and C-reactive protein (CRP) [72]. A meta-analysis of nine different studies demonstrated significantly higher IL-6 levels in people with severe COVID-19 disease than with non-severe disease (mean difference: 38.6 pg/mL, 95% CI: 24.3–52.9 pg/mL) and was associated with excess mortality [73].

Binding of SARS-CoV-2 to alveolar epithelial cells activates the immune system, leading to secretion of a large number of cytokines, including IL-6 [74]. IL-6 is an important pro-inflammatory and multifunctional cytokine involved in acute inflammation, metabolism, and autoimmune cell differentiation [75]. IL-6 binds to soluble forms of IL-6 receptor (sIL-6R) and forms a complex with gp130 dimers on the cell surface, activating the Janus kinase-signal transducer and activator of transcription 3 (JAK-STAT3) signaling and inducing CRS [74]. Subsequently, vascular endothelial growth factor (VEGF), MCP-1, IL-8, and more IL-6 are secreted, while E-cadherin is downregulated on endothelial cells [76]. The reduction of E-cadherin and VEGF overexpression give rise to increased vascular permeability [77]. IL-6 activates B cells, enhances the expansion and proliferation of T cells, regulates CTL activity, promotes Th17 cell differentiation and inhibits the activation of regulatory T cells (Tregs) [78]. Tocilizumab is a recombinant humanized monoclonal antibody against human IL-6R [79,80]. It has been approved for the treatment of rheumatoid arthritis and systemic juvenile idiopathic arthritis and there is increasing evidence it may be useful for COVID-19 disease [81,82,83].

Clinical analysis of COVID-19 patients admitted to a hospital in Wuhan, China reported that patients showed elevated plasma concentrations of other cytokines including TNF-α, IL-7, IL1β, INFγ, IL1RA and MIP1A/B. Moreover, cytokine levels were higher in patients admitted to the intensive care unit (ICU) [84]. Tumor necrosis factor-α (TNF-α) is an inflammatory cytokine secreted by activated monocyte/macrophages as part of innate immune system and is directly correlated with COVID-19 disease severity (*R* = −0.322, *p* < 0.001) [85]. Although targeting TNF-α is rational and appealing, the clinical efficacy of anti-TNF antibodies (e.g., adalimumab) for SARS-CoV-2 infected patients needs further investigation [86].

## 7. COVID-19 and Comorbidities

The clinical presentations of COVID-19 are heterogeneous, so identifying comorbidities associated with COVID-19 is important for several reasons. First, when treating patients, it allows health care providers to adjust and select the best treatment option for vulnerable patients, reducing the risk of side effects due to polypharmacy or drug–drug interactions. Second, it identifies people at higher risk, assisting governments to stratify public health policies and guidelines to allow easing of social distancing restrictions, while protecting the vulnerables. Finally, determining which comorbidities are most associated with poor COVID-19 outcomes will help researchers to understand the pathophysiology of SARS-CoV-2 infection and its interactions with underlying chronic diseases.

Early clinical experience suggested that the elderly and people of any age with serious underlying medical conditions are at a greater risk of severe illness from COVID-19 [87]. Consistent with this, previous studies showed the presence of comorbidities conferred a 3–4 fold increased risk of developing acute respiratory distress syndrome in patients with SARS-CoV [88] and MERS-CoV infections [89]. For COVID-19, medical conditions that put people at increased risk of severe disease include: uncontrolled diabetes; hypertension; lung, liver, and kidney disease; cancer; smoking; solid organ transplant; pregnancy; thalassemia; asthma; neurologic conditions, such as dementia; pulmonary fibrosis (having damaged or scarred lung tissues); obesity and long term steroid therapy [90,91]. A systematic review and meta-analysis of 1576 patients with COVID-19 showed that hypertension (21.1%) and diabetes (9.7%) were the most prevalent comorbidities, followed by cardiovascular disease (8.4%) and respiratory disease (1.5%) [92]. This reflects international studies, which show that comorbidities associated with poor outcomes in COVID-19 include hypertension (20–30%), diabetes (10–20%), cardiovascular disease (8–12%), chronic obstructive pulmonary disease (COPD) (1.5–7.5%), chronic kidney diseases (1–3%), cerebrovascular disease (1.5–3%), co-infection with human immunodeficiency virus (HIV) or hepatitis B virus (1–2%), malignancy (1–3.5%), respiratory illnesses (1.4%), renal disorders (0.5–1.5%) and immunodeficiencies (0.01%) [93,94,95,96,97,98]. The most common comorbidity varied among the seven countries reviewed, as follows: China (hypertension 39.5%), South Korea (cardiovascular disease 25.6%), Italy (hypertension 35.9%), USA (hypertension 38.9%), Mexico, (other 42.3%), UK (hypertension 27.8%), Iran (diabetes 35.0%); diabetes was the second most common comorbidity in five of them [93]. Evaluating the mechanisms underlying the associations of severe COVID-19 disease with hypertension, diabetes and respiratory diseases is crucial for the management of high-risk patients and for developing policies and guidelines to reduce future risk of severe COVID-19 disease. Finally, strong precaution should be considered when managing and treating COVID-19 patients with preexisting cardiovascular risk factors, especially high blood pressure and diabetes, while patients with uncontrolled hypertension should be informed about their increased risk and advised to take appropriate preventative measures.

## 8. Available Treatment Options for COVID-19

**Figure 2:**
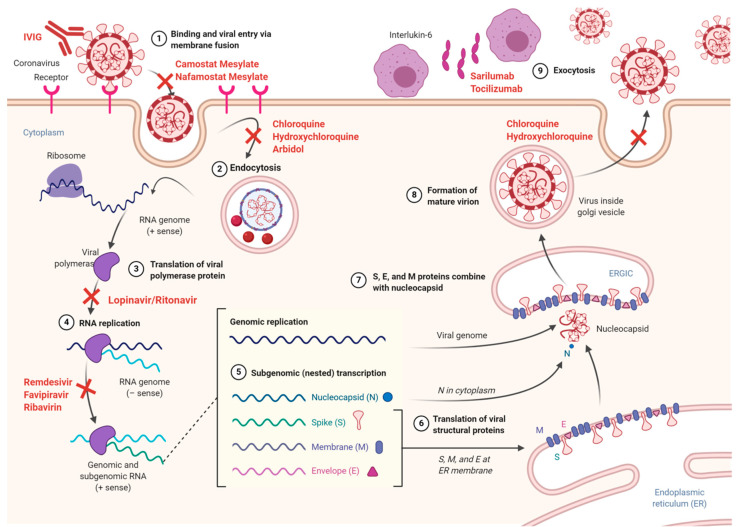
The life cycle of SARS-CoV-2 and potential mechanisms of action of drugs. The virus lifecycle starts when the S protein binds to its cellular receptor angiotensin converting enzyme II (ACE2). Following a conformation change in the S protein, the viral envelope fuses with the cell membrane. After endocytosis, the RNA of SARS-CoV-2 is released to the cytoplasm. Genomic RNA, which is positive sense, is translated into the viral polyproteins pp1a and 1ab. The polymerase produces a series of subgenomic mRNAs and translates the relevant viral proteins. In the endoplasmic reticulum (ER) and Golgi, viral proteins and genome RNA are assembled into virions and transported via vesicles and released out of the cell. ERGIC (ER–Golgi intermediate compartment). Site of action for each therapeutic candidate is shown with red cross.

An array of disease modifying drugs have been proposed for the specific treatment of COVID-19 disease and will be discussed below. The classification of drugs and the effective doses are listed in Table 1 while their mechanism of action is illustrated in Figure 2.

### 8.1. Antivirals

#### 8.1.1. Remdesivir

Remdesivir (RDV) was originally developed as a treatment for Ebola and Marburg virus infection and is a promising candidate for treatment of COVID19. RDV is an adenosine nucleotide analogue with broad-spectrum antiviral activity against RNA viruses including *Filoviridae, Paramyxoviridae, Pneumoviridae,* and *Coronaviridae.* It acts as an inhibitor of RdRps and inhibits virus replication through premature termination of RNA transcription as it incorporates into nascent viral RNA chains [99]. Antiviral activity of RDV against coronaviruses including SARS-CoV and MERS-CoV was described in 2017 [100]. Recently, an in vitro study on Vero E6 cells has shown that remdesivir at low-micromolar concentrations blocks SARS-CoV-2 infection with high selectivity (half-maximal effective concentration (EC50), 0.77 μM; half-cytotoxic concentration (CC50) > 100 μM; SI > 129.87) [101]. Interestingly, remdesivir demonstrated promising results in treatment of the first patient diagnosed with COVID-19 in the United States [102]. In order to further investigate the efficacy and safety of this drug, four clinical trials in severe and mild/moderate respiratory infections by SARS-CoV-2 in the United States and two in China have been commenced [103]. The first randomized controlled clinical trial of remdesivir in the United States, known as the Adaptive COVID-19 Treatment Trial, enrolled 1063 patients and showed that patients who received remdesivir recovered faster, with a median time of 11 rather than 15 days (*p* < 0.001). There was also a non-significant trend towards reduced mortality (8.0% in remdesivir arm vs 11.6% for the placebo arm, *p* = 0.059) [104]. In another cohort of 53 patients hospitalized for severe COVID-19 in the United States, Europe, Canada or Japan a 10-day course of remdesivir resulted in clinical improvement in 36 patients (68%) [105]. Another randomized, double-blind, placebo-controlled, multicenter trial of remdesivir in 237 laboratory-confirmed SARS-CoV-2 infected patients, from ten hospitals in China (NCT04257656), revealed that time to clinical improvement was not associated with the use of remdesivir; adverse events were reported in 66% of patients [106]. On May 1st the emergency use of remdesivir for adults and children hospitalized with severe suspected or laboratory-confirmed COVID-19 was approved by the US FDA [4].

#### 8.1.2. Lopinavir/Ritonavir

Lopinavir*/*Ritonavir (LPV/r) is a coformulation of two structurally related protease inhibitor antiretroviral agents primarily used for treatment of HIV-1 infection in adults and children over 2 years of age. LPV/r reduces the replication of SARS-CoV and MERS-CoV in vitro [107,108] by inhibiting viral proteases including 3CLpro or PLpro [108]. Ritonavir is used in combination with Lopinavir to increase LPV half-life by inhibiting Cytochrome P450 3A4 (CYP3A4) [109]. A study of six patients with confirmed COVID-19 infection in Korea and China showed a significant decrease in SARS-CoV-2 viral load and clinical improvement after administration of LPV/r [110,111]. However, concomitant drug therapies, variation in starting time of therapy, different levels of illness severity, and the lack of a control group reinforces the need for caution when interpreting such results [80,110,111]. In another study in Singapore the efficacy of LPV/r was evaluated on five patients with COVID-19 who required supplemental oxygen. Three of them improved within 3 days, while two experienced progressive respiratory failure. [112]. On the other hand, in a retrospective cohort study among 29 hospitalized patients who received LPV/r and were discharged, no differences in viral shedding duration was noted [113]. Similarly, Cao et al. reported a clinical trial including 199 patients with SARS-CoV-2 infection, which showed that LPV/r was not associated with clinical improvement [114]. However, the low efficacy of LPV/r in this study might be attributed to the advanced pneumonia in treated patients coupled with a lack of knowledge about the exact concentrations required to inhibit viral replication [115]. So far 10 ongoing clinical trials have been registered from China, Korea, Thailand and Hong Kong to evaluate efficacy of LPV/r for the treatment of COVID-19, either alone or in combination with other antivirals including ribavirin, interferon beta-1b or Chinese traditional medicine [103]. It is important to note that LPV/r has significant drug interactions including the risk of cardiac arrhythmia due to QT- prolongation [116]. Cao et al. documented multiple side effects of LPV/r including gastrointestinal adverse events (i.e., anorexia, nausea, abdominal discomfort, or diarrhea) as well as acute gastritis, hepatic injury risk, pancreatitis, more severe cutaneous eruptions, and QT prolongation, and the potential for multiple drug interactions [114]. The European Society of Intensive Care Medicine (ESICM) and the Society of Critical Care Medicine (SCCM) have recommended against the routine use of LPV/r in critically ill COVID-19 patients [117]. The National Institutes of Health (NIH) guidelines recommend against the use of Lopinavir/ritonavir as a clinical benefit has not been demonstrated in patients with COVID-19, due to unfavorable pharmacodynamics and a lack of clinical trials [2].

#### 8.1.3. Favipiravir

Favipiravir (FPV) is a guanine nucleic acid analogue approved for the treatment of resistant strains of influenza [118]. Intracellular phosphoribosylation of favipiravir generates the active form favipiravir-RTP (favipiravir ribofuranosyl-5B-triphosphate) which is a substrate for the RNA-dependent RNA polymerase of RNA viruses and inhibits polymerase activity [118]. Favipiravir suppresses the replication of flavi-, alpha-, filo-, bunya-, arena-, noro-, and of other RNA viruses [119]. Therefore, favipiravir may also have activity against SARS-CoV-2. The use of favipiravir for COVID-19 has been reported from limited clinical experiences. An open-label study in China compared the effects of favipiravir plus interferon-α with LPV/r plus interferon-α for the treatment of COVID-19. Favipiravir was associated with more rapid viral clearance, better improvement in chest imaging results and less adverse effects [120]. Based on these results favipiravir was approved as the first anti-COVID-19 drug in China [121]. Another randomized clinical trial compared the effect of favipiravir and arbidolol in COVID-19 patients but demonstrated no difference in clinical recovery rate at day 7. However, favipiravir significantly shortened the latency to relief from pyrexia and cough [122]. An ongoing clinical trial (NCT04303299) is investigating the efficacy, performance and safety of favipiravir in combination with oseltamivir and chloroquine among 80 participants with COVID-19 in Rajavithi Hospital in Thailand; results will be released in November 2020 [123]. There is less preclinical support for favipiravir to treat COVID-19 compared with remdesivir, due to its higher EC50 (61.88 μM) compared to remdesevir (0.77 μM) [101,124]. Favipiravir is well tolerated in healthy volunteers, however caution is required as it is teratogenic [119].

#### 8.1.4. Ribavirin

Ribavirin (1-β-D-ribofuranosyl-1,2,4-triazole-3-carboxamide) is a synthetic guanosine analogue with broad-spectrum activity against several RNA and DNA viruses [125]. It was first approved in 1972 and has been used as a therapeutic agent against respiratory syncytial virus, Lassa fever virus, influenza A and B and in combination with interferon-α for hepatitis C [126]. Ribavirin has multiple mechanisms of action-including interference with RNA capping, inhibition of polymerases, introducing random mutations, suppression of natural guanosine generation by inhibition of inosine monophosphate dehydrogenase and immunomodulatory effects by enhancing the T cell response [127]. The in vitro anti SARS-CoV activity reported for ribavirin differs widely depending on the cell lines used for the antiviral assays. It is possible that ribavirin may be more effective when co-administrated with IFN as this combination inhibited replication of SARS-CoV in five different cell types of animal or human origin, at considerably lower concentrations than with either single treatment alone [128]. Similarly, the combination of IFN-α2b and ribavirin in vitro decreased the inhibitory concentration against MERS-CoV to levels that are likely achievable in humans [129]. In terms of SARS-CoV-2, in vitro studies revealed that high concentrations of ribavirin (EC50 of 109.5 µM) are required to reduce the viral infection and ribavirin is 100 times less potent than remdesivir [101]. Administration of high doses of ribavirin in SARS trials was associated with serious adverse reactions including haemolytic anaemia, electrolyte disturbances and significant teratogenicity. [88,130,131]. Given its considerable adverse effect profile, toxicity and the lack of in vitro data evaluating the efficacy of this compound, the administration of ribavirin for the treatment of COVID-19 is not recommended. An ongoing trial in China (ChiCTR2000029387) is evaluating the efficacy and safety of ribavirin and LPV/r in combination with interferon alpha-1b for SARS-CoV-2 infection [132].

#### 8.1.5. Arbidol Hydrochloride (Umifenovir)

Arbidol (ARB; ethyl-6-bromo-4-[(dimethylamino) methyl]-5-hydroxy-1-methyl-2-[(phenylthio)methyl]-indole-3-carboxylate hydrochloride monohydrate), an indole derivative, is a broad-spectrum antiviral against various enveloped and non-enveloped viruses and is widely used in Russia and China [133]. Arbidol displays antiviral activity in vitro and/or in vivo against influenza viruses A, B and C, adenovirus, respiratory syncytial virus, SARS-CoV, parainfluenza type 5, poliovirus, rhinovirus 14, coxsackievirus B5, hantavirus, Chikungunya virus, hepatitis B virus (HBV) and hepatitis C virus (HCV) [134]. The anti-viral mechanism of arbidol is thought to relate to inhibition of viral fusion with target cell membranes and with the membranes of endosomes and consequently blockage of virus entry [133]. For influenza A and B viruses it targets hemagglutinin (HA), the major glycoprotein on the surface of the virion, thereby preventing infection [135]. For SARS-CoV, studies have shown direct antiviral effects of arbidol and arbidol mesylate in vitro, with arbidol mesylate reducing the reproduction of SARS-CoV five times more effectively than arbidol [136]. In China, limited clinical experience with arbidol for COVID-19 treatment has been described. In one study from Wuhan in 67 hospitalized patients with confirmed SARS-CoV-2 infection, 36 (53.7%) received arbidol treatment for a median duration of 9 days and demonstrated lower mortality rates (0% (0/36) vs. 16% (5/31)) and higher discharge rates (33% (12/36) vs. 19% (6/31)) compared with patients who did not receive this drug [137]. In a prospective, randomized, controlled, open-label multicenter trial investigating the efficacy of favipiravir compared with arbidol in the treatment of COVID-19 patients, the clinical recovery rate at day 7 did not differ between the favipiravir and arbidol groups [122]. Currently, multiple clinical trials are investigating the efficacy of arbidol on COVID-19 patients in China and USA (NCT04260594, NCT04255017). Four clinical trials are comparing arbidol with oseltamivir, LPV/r and carrimycin for COVID-19 [123].

#### 8.1.6. Camostat Mesylate (FoipanTM)

Camostat mesylate (*N*,*N*-dimethylcarbamoylmethyl 4-(4-guanidinobenzoyloxy)-phenylacetate) is a synthetic serine protease inhibitor used for the treatment of chronic pancreatitis, dystrophic epidermolysis, oral squamous cell carcinoma and exocrine pancreatic enzyme inhibition [138]. Camostat mesylate was developed in Japan in the 1980s but very little data from outside Japan is available. Some coronaviruses (SARS-CoV, MERS-CoV) and influenza virus activate their envelope glycoproteins by employing host cell proteases [139]. Membrane fusion and host cell entry of SARS-CoV is mediated by spike protein cleavage and activation which requires TMPRSS2, an airway and alveolar cell serine protease [140]. Zhou et al. demonstrated that serine proteases cause the spread of SARS-CoV and camostat effectively prevents this step, suggesting it may be an option for treating SARS and potentially MERS [141]. Another study demonstrated that camostat partially blocked SARS-CoV and human coronavirus NL63 (HCoV-NL63) infection in HeLa cells expressing the ACE2 receptor and TMPRSS2 [142]. Additionally, the effect of camostat mesylate in blocking SARS-CoV-2 infection of human lung Calu-3 cells has been demonstrated [143]. A double-blind randomized controlled clinical trial is evaluating the efficacy of camostat mesylate for treatment of COVID-19 (NCT04353284), but more studies are needed to clarify whether adequate concentrations for suppressing the virus can be attained in a patient’s lung.

#### 8.1.7. Nafamostat Mesylate

Nafamostat mesylate (6-amidino-2-naphthyl p guanidinobenzoate dimethanesulfonate) is a synthetic serine protease inhibitor available in Japan since 1986 that has been approved for the treatment of inflammatory-related diseases such as pancreatitis [144,145]. Nafamostat is a potent inhibitor of S-mediated membrane fusion of MERS-CoV and blocks MERS-CoV infection in vitro [22]. The anti MERS-CoV activity of nafamostat is through inhibition of the host protease TMPRSS2, which is responsible for the proteolysis of the S protein after the receptor-binding stage [22]. Recently a study in lung cell culture tested the inhibitory effect of gabexate mesylate, nafamostat mesylate and camostat mesylate on SARS-CoV-2 infection and demonstrated that nafamostat mesylate inhibited S-mediated entry of SARS-CoV-2 into host cells 15 times more effectively that camostat mesylate, with an EC50 in the low nanomolar range [146]. In another study, nafamostat was tested in vitro against a clinical SARS-CoV-2 isolate and was shown to have antiviral activity, with an EC50 of 22.50 μM [101]. An advantage of targeting host proteases instead of viral protein inhibitors is that it avoids treatment failure due to mutations of the target. Despite these promising in vitro data, the efficacy of nafamostat as a COVID-19 treatment needs to be tested in clinical trials.

### 8.2. Anti-Parasites

#### 8.2.1. Chloroquine/Hydroxychloroquine

Chloroquine (*N*4-(7-Chloro-4-quinolinyl)-*N*1,*N*1-diethyl-1,4-pentanediamine) has long been used to prevent and treat malaria. It has also been tested in chronic viral diseases and has shown antiviral activity against RNA and DNA viruses including HIV, influenza A and B viruses, influenza A H5N1 virus, HCV, rabies virus, hepatitis A virus, poliovirus, Chikungunya virus, Lassa virus, Dengue virus, Hendra and Nipah viruses, Crimean–Congo hemorrhagic fever virus, Zika virus and Ebola virus, as well as HBV and herpes simplex virus [147]. Sometimes the antiviral activities of chloroquine described in vitro have been confirmed in virus-infected patients, however, the results have not always been reproduced in clinical trials.

Chloroquine possesses different mechanisms of action depending on the pathogen studied. In vitro studies on SARS-CoV showed that chloroquine can interfere with glycosylation of ACE2, in Vero cells [148]. Since SARS-CoV-2 uses a similar receptor, it is expected that chloroquine may prevent SARS-CoV-2 attachment to target cells by the same mechanism. Moreover, chloroquine can induce autophagosome formation resulting in spike protein degradation by changing the pH of lysosomes and inhibition of cathepsins. Furthermore, chloroquine interferes with viral assembly, budding and proteolytic processing of the M protein through inhibition of Mitogen-activated protein kinases (MAPK) [149].

Previous studies have reported the potential use of chloroquine for treatment of SARS-CoV [150]. The potent efficacy of remdesivir and chloroquine were evaluated in Vero E6; chloroquine was effective in controlling SARS-CoV-2 infection in vitro with an EC50 at 48 h of 1.13 μM [101], comparable with previous in vitro findings against SARS-CoV and MERS-CoV [151]. Besides the antiviral activity of chloroquine, it has immune modulatory functions by inhibition of TNF-α and IL-6 production and secretion. Upon oral administration of chloroquine, it is widely distributed systemically in the body, reaching the lung [101]. There is safety concern in administration of chloroquine due to the risk of macular retinopathy and cardiomyopathy [152,153]. Despite the in vitro efficiency of chloroquine, the clinical outcomes are uncertain.

Hydroxychloroquine sulfate, a derivative form of chloroquine, was first synthesized in 1946 by introducing a hydroxyl group into chloroquine. It has two to three times less toxicity in animals compared to chloroquine [154]. Hydroxychloroquine likely has the same mechanism of action on viruses as that of chloroquine and acts as a weak base compound, changing the pH of endosomes/lysosomes [155].

There is limited published evidence supporting the activity of hydroxychloroquine against coronaviruses. An in vitro study in Vero cells showed that the chloroquine was approximately 5-fold more potent (EC50 of 6.5 ± 3.2 μM) than that of hydroxychloroquine (EC50 of 34 ± 5 μM) against SARS-CoV [156]. Conversely, the potency of hydroxychloroquine in Vero cells infected with SARS-CoV-2 (EC50 of 0.72 μM) was greater than that of chloroquine (EC50 of 5.47 μM) [157]. In a non-randomized open-label trial in France, administration of hydroxychloroquine significantly reduced the viral load in COVID-19 patients, alone or in combination with macrolide antibiotics [158]. However, there are some limitations to the study including small sample size, unclear criteria for enrollment of cases and controls, lack of clinical outcomes as well as enrolment of asymptomatic individuals. Widespread administration of hydroxychloroquine is likely to cause rare adverse effects in patients, including fulminant hepatic failure, serious cutaneous adverse reactions and ventricular arrhythmias (especially when administrated with azithromycin) [159]. It has been shown the combination of hydroxychloroquine and azithromycin in COVID-19 patients is associated with increased risk of QTc prolongation, cardiac dysrhythmias, and death [160,161]. Additionally, the FDA does not recommend combination therapy of remdesivir with chloroquine phosphate or hydroxychloroquine sulfate as it might result in a reduced antiviral activity of remdesivir [162]. Moreover, the FDA announced the use of hydroxychloroquine and chloroquine for treatment of hospitalized COVID-19 patients can cause serious heart rhythm problems and other safety issues, including blood and lymph system disorders, kidney injuries, liver problems and even liver failure [163]. Owing to the potential side effects—namely heart problems—on June 15th the FDA withdrew the emergency use authorization it issued in March for chloroquine and hydroxychloroquine as COVID-19 treatments. The FDA made this determination based the results from the RECOVERY trial in 1542 hospitalized COVID-19 patients administrated hydroxychloroquine, which found no significant difference in the primary endpoint of 28-day mortality or hospital stay duration [163,164].

At least 16 different trials for SARS-CoV-2 are already registered in the Chinese clinical trial registry (http://www.chictr.org.cn) testing the efficacy of chloroquine and hydroxychloroquine. In addition, hydroxychloroquine is now under evaluation in the U.S. (NCT04308668) as a post-exposure prophylaxis/preemptive therapy for SARS-CoV-2 infection. Recently the NIH guidelines panel for COVID-19 treatment recommended against the use of chloroquine or hydroxychloroquine for the treatment of COVID-19, except in a clinical trial. The panel also recommends against the use of high-dose chloroquine (600 mg twice daily for 10 days) for the treatment of COVID-19 [165].

#### 8.2.2. Ivermectin

Ivermectin is an FDA-approved broad spectrum anti-parasitic agent mainly used in the treatment of onchocerciasis, which shows anti-viral effects against HIV-1, simian virus SV40, dengue virus (DENV), West Nile Virus, Venezuelan equine encephalitis virus (VEEV) and Influenza virus [166]. Ivermectin inhibits the interaction between the integrase protein (IN) of HIV-1 and importin (IMP) α/β1 heterodimer that is responsible for IN nuclear import [167]. Due to the important role of nuclear transport of viral proteins in the replication cycle as well as inhibition of the host antiviral response, targeting this step might be a therapeutic approach [168]. Recently, a study on Vero-hSLAM cell lines infected with SARS-CoV-2 has shown that ivermectin can reduce viral RNA up to 5000-fold, equal to a 99.98% reduction after 48 h [166]. Momekov et al. using available pharmacokinetic data from ivermectin showed that its inhibitory concentrations against the SARS-CoV-2 is unlikely attainable in humans [169]. Overall, the possible efficacy of ivermectin against SARS-CoV-2 infection needs data to support it.

## 9. Immunotherapy

Several studies have been undertaken to develop new treatment strategies for viral infections and the use of immunotherapy is encouraging [170]. One of the most useful immunotherapy methods is passive immunity [171], i.e., the transfer of active humoral immunity including ready-made antibodies. There are two main categories of acquired artificial passive immunity against COVID-19: treatment with monoclonal antibodies (mAbs) and convalescent plasma from people recovering from COVID-19 [171]. The antibodies directly bind to the viral antigen and provide an effective anti-viral response. Several monoclonal antibodies are currently being studied or are undergoing clinical trials [172].

### 9.1. Convalescent Plasma Therapy

Convalescent plasma therapy relies on the administration of plasma from recovered COVID-19 patients. Previous coronavirus outbreaks including SARS-CoV and MERS-CoV as well as Ebola virus disease, avian influenza A (H5N1), 2009 H1N1 pandemic influenza A, respiratory syncytial virus, Zika viruses, human cytomegalovirus and rabies have confirmed the presence of neutralizing antibodies in convalescent serum [173,174]. However, convalescent plasma was not successful for Ebola [175].

Patients with SARS-CoV-2 infection develop a serum antibody response (IgG) to different epitopes of the virus and some of these antibodies have the potential to neutralize the virus. To prepare convalescent plasma, plasma rich in IgG antibody is collected at least two weeks after the recovery date [176]. In SARS-CoV-2, passive antibody therapy mediates protection by virus neutralization and perhaps by phagocytosis and/or antibody-dependent cellular cytotoxicity [177]. A study by Duan et al. [3] of 10 adult patients with severe COVID-19 demonstrated that a single dose (200 mL) of convalescent plasma was not only well tolerated but was able to increase and maintain neutralizing antibodies at a high level. Moreover, clinical and paraclinical symptoms improved within three days and viral RNA was undetectable in blood seven days after the transfusion [3]. Passive antibody therapy is more effective as prophylaxis than for treatment and in infected patients its effectiveness was higher when administered shortly after the appearance of symptoms [177].

In a Hong Kong study, 80 SARS patients who were administrated convalescent plasma before day 14 of disease showed an improved prognosis and a higher discharge rate (58.3% vs. 15.6%), including those who were polymerase chain reaction (PCR) positive and seronegative for SARS-CoV at the time of therapy [178]. Similarly, transfusion of 500 mL convalescent plasma from three convalescent SARS patients to three health care workers with SARS infection in Taiwan resulted in survival of all three patients, a decrease in viral loads to 0 or 1 copy/mL one day after transfusion and increasing levels of anti-SARS-CoV IgM and IgG [179]. However, administration of convalescent plasma to three patients with MERS in South Korea showed the presence of neutralizing antibodies in only two of them [180]. Concerning COVID-19, Zhang et al. demonstrated anti-SARS-CoV-2 activity of convalescent plasma from six patients in China who had recovered; transfusion of the plasma to a patient with severe COVID-19 resulted in clinical improvement, with no requirement for mechanical ventilation 11 days post-plasma transfusion [181]. Similarly, four critically ill COVID-19 patients who received convalescent plasma and supportive care recovered [104]. The US FDA has approved clinical trials to test the efficacy and safety of plasma from patients recovering from COVID-19 [182,183,184,185,186,187,188,189], joining other registered trials in China, Columbia, Iran, Mexico and Baylor Research Institute in USA [182,183,184,185,186,187,188,189].

### 9.2. Intravenous Immunoglobulin (IVIG)

IVIG is a supportive therapy prepared from pooled plasma of healthy humans. IVIG is traditionally used as replacement therapy in patients with humoral immune deficiencies [190]. Unlike convalescent plasma, IVIG does not contain a SARS-CoV-2 neutralizing antibody [191]. IVIG has various mechanisms of action including interference with B-cell antigen presentation, immune-modulation and immune substitution [192]. During the SARS outbreak, IVIG was used as a therapy, but evidence supporting its effectiveness is inconclusive [193]. Despite some reports on the use of IVIG in MERS infection, there is not any evidence of anti-MERS activity [194]. As these antibodies are administered intravenously, they can cause renal failure or thrombosis in MERS patients [195]. For COVID-19, Shi et al. reported successful treatment of a confirmed patient with advanced respiratory failure, shock and persistent diarrhea, using intensive plasma exchange (PE) and IVIG transfusion [196]. They emphasized the importance of early initiation of PE treatment and IVIG to avoid progression to ARDS and multi-organ failure. Similarly, a multicenter retrospective cohort study of 325 critically unwell adult COVID-19 patients in China who were exposed to IVIG, demonstrated that although early administration improved their prognosis, mortality at 28-days and 60-days was not improved and total duration of disease was longer [197]. In a retrospective study of 58 severe or critical COVID-19 patients in Wuhan treated with IVIG, administration of IVIG within 48 h of admission to ICU improved prognosis by reducing mechanical ventilation requirement and length of stay [198]. It is important to note that all of these studies were carried out in the context of small sample size and multiple drug treatments; thus, the efficacy and the associated adverse effects of IVIG remain unclear. In order to evaluate the efficacy and safety of IVIG therapy in patients with severe or critical COVID-19, a single-center randomized, open-label, controlled study has been registered (NCT04261426). Like other proposed treatments for COVID-19, IVIG is still unlicensed and insufficient evidence exists to support its use in the management of COVID-19.

### 9.3. Monoclonal Antibodies (mAbs)

Monoclonal antibodies are synthesized by one unique parental cell and thus bind to a unique epitope. To date, several mAbs have been approved by the FDA for viral infections including SARS-CoV-2 [199]. IL-6 is an attractive target for mAb therapy due to its role in cytokine storm mediated lung inflammation [200], body temperature regulation and fever. In a study on 20 patients with COVID-19 in Anhui, China more than 75% of patients admitted to ICU who were given tocilizumab showed improvement in oxygen saturation and 90% reported resolution of lung opacities on CT scans, while CRP decreased in 84% [80]. In order to compare the efficiency of tocilizumab and favipiravir, a randomized controlled trial has been registered in China (ChiCTR2000030894) [201]. Sarilumab (Kevzara) is another IL-6 receptor antagonist (recombinant humanized anti IL-6R monoclonal antibody) approved for treatment of rheumatoid arthritis [202]. It is currently being evaluated in a Phase 2/3 clinical trial enrolling hospitalized adult COVID-19 patients, sponsored by Sanofi and Regeneron Pharmaceuticals (NCT04315298).

Another inflammatory cytokine involved in COVID-19 disease is TNF-α, which can be targeted by mAbs like adalimumab. Currently, there are several trials registered for assessment of adalimumab or biosimilar mAbs (Qletli) for their safety and efficacy in COVID-19 treatment in China (ChiCTR2000030089, ChiCTR2000030580). However, upper respiratory tract infections and reactivation of hepatitis B infection are common and concerning side effects [203].

Baricitinib is an orally administered, JAK antagonist monoclonal antibody used routinely for the treatment of rheumatoid arthritis [204]. Although JAK blockers are considered as potential therapeutic agents in inflammatory storms, several researchers have reported that baricitinib is unlikely to be useful for COVID-19 [205] due to its well-known side effects, including lymphocytopenia and neutropenia, and therefore the risk of viral reactivation [205].

Two other potential mAbs are Leronlimab and Ibalizumab, both of which bind specifically to the C–C chemokine receptor type 5 (CCR5). A phase II clinical trial assessing leronlimab in COVID-19 patients with respiratory complications was registered in April 2020 [206]. Ibalizumab is non-immunosuppressive, FDA approved for the treatment of multidrug-resistant HIV in 2018 [207] and may be considered as a co-treatment agent for COVID-19 in the future.

Camrelizumab is a humanized anti programmed death-1 (PD-1) monoclonal antibody. Blocking PD-1 signaling rescues exhausted CD8+T cells and restores their activity during viral infections, but lung toxicity is reported [208]. A clinical trial has been registered using camrelizumab for treatment of critical COVID-19 patients in China [209]. Omalizumab is an IgG1 humanized anti IgE monoclonal antibody that reduces hypersensitivity to allergens and also decreases late inflammation events, so may be considered for COVID-19 patients with dermatologic symptoms [210].

Granulocyte-monocyte colony stimulating factor (GM-CSF) is another immunomodulatory cytokine reported to be present at increased levels in COVID-19 patients [84]. In one clinical trial, adding a single dose of mAb targeting GM-CSF (mavrilimumab) to standard hospital treatment improved clinical outcomes in patients with COVID-19 [211].

The mAbs mentioned above were used to modulate the immune response, but specific human monoclonal antibodies can also be used to neutralize SARS-CoV-2, for both treatment and prevention of COVID-19. Several neutralizing monoclonal antibodies against SARS-CoV and MERS-CoV have potential cross reactivity against SARS-CoV-2 [212]. For example, recent studies showed that a monoclonal antibody (S309) derived from memory B cells from an individual convalescing from SARS-CoV infection are also capable of neutralizing SARS-CoV-2 [213].

By screening hybridomas from immunized mice using pseudotyped vesicular stomatitis virus, a chimeric mAb called 47D11 was identified which has cross-neutralizing activity against the S protein from SARS-CoV and SARS-CoV-2. This antibody was subsequently cloned to produce a fully humanized mAb, which in cell culture was able to block SARS-CoV-2 infection [214]. In another study, four mAbs were isolated from individuals who recovered from COVID-19 (B5, B38, H2, and H4), which were able to neutralize SARS-CoV-2 effectively. In particular, B38 and H4 were able to bind RBD of the viral S protein, inhibiting its attachment to ACE2 and reducing the virus titer in animal models [215].

## 10. Adjunctive/Supportive Therapy

### 10.1. Azithromycin

Azithromycin (9-deoxo-9a-aza-9a-methyl-9a-homoerythromycin) is a widely used macrolide antibiotic which can inhibit protein synthesis in bacteria by targeting 23S ribosomal RNA. Beside its antibacterial activity, azithromycin has immunomodulatory and anti-viral effects. Previous studies have suggested an antiviral function for azithromycin in Ebola virus infection, both in vitro and in mice [216]. Azithromycin (but not other macrolides like erythromycin or telithromycin) can inhibit rhinovirus replication and release in human bronchial epithelial cells, by inducing expression of interferon and pro-inflammatory cytokines (IL-6 and IL-8) [217]. Azithromycin also shows dose dependent antiviral activity against Zika virus in several cell lines (Vero, Hela, A549, Huh7), inhibiting late stages of virus replication by enhancing the type I and III interferon responses [218]. Interestingly, Tran et al. reported antiviral activity of azithromycin against Influenza (H1N1) virus; treatment prior to infection inhibited virus replication by blocking internalization into host cells, without changing viral attachment [219]. Moreover, intranasal administration of azithromycin to infected mice reduced the viral load in affected lungs [219].

In a non-randomized clinical trial in COVID-19 patients (*n* = 36), Gautret et al. reported that azithromycin co-administered with hydroxychloroquine reduced the viral load more efficiently than hydroxychloroquine alone [158]. Patients were administered hydroxychloroquine (600 mg/day), with or without azithromycin (500 mg day one, followed by 250 mg/day for four days) and SARS-CoV-2 was measured in nasopharyngeal swabs by quantitative reverse transcriptase PCR (qRT-PCR). They found that at day 6 all of the patients in the combination group (6/6) were virologically cured (negative swab), compared to hydroxychloroquine alone (57.1%) or untreated controls (12.5%) [158]. However, this small trial was not randomized, patients were not well matched and adding azithromycin to hydroxychloroquine may increase the risk of cardiac arrhythmias. There are now several larger clinical trials investigating the efficacy of azithromycin in COVID-19 patients (e.g., NCT04332107, NCT04336332, NCT04329832), but until these results are available, Azithromycin cannot be recommended for routine use in COVID-19.

### 10.2. Corticosteroids

Corticosteroids are one of the main class of drugs used for chronic inflammatory diseases and immune disorders. Their anti-inflammatory effects are attributed to activating the transcription of anti-inflammatory genes and suppressing pro-inflammatory genes through modulation of histone acetylation [220]. For pneumonia, systemic corticosteroids can be used as an adjuvant to antibiotics, with an analysis of randomized control studies showing that corticosteroids reduce both mortality and morbidity in adults with severe community acquired pneumonia [221].

Inflammatory responses of host immune cells and the resulting cytokine storm are two important aspects of the pathogenesis in severe SARS-CoV-2 infection [222]. However, a meta-analysis of previous observational studies revealed that corticosteroid therapy is associated with increased mortality (Odds Ratio OR = 2.12) in patients with influenza [223] and can delay viral clearance in critically ill MERS patients (adjusted hazard ratio = 0.35) [224]. Another meta-analysis of 15 studies in coronavirus infected patients (including SARS-CoV, SARS-CoV-2 and MERS-CoV) revealed that while corticosteroids are likely to be used in critically ill patients, treatment was associated with increased mortality (Risk Ratio RR = 2.11, *p*-value = 0.019), increased bacterial infection (RR = 2.08, *p* < 0.001) and led to longer hospital stays (total number of patients = 5270) [225]. In an observational study of SARS-CoV-2 infected patients treated with 40 mg methylprednisolone once or twice daily, there was no significant association between corticosteroid treatment and time to viral clearance or length of hospitalization [226]. Similarly, Fang et al. reported that low-dose methylprednisolone by injection (median hydrocortisone-equivalent dose, 250 mg/day) or orally (median hydrocortisone-equivalent dose = 237.5 mg/day) had no significant impact on SARS-CoV-2 viral clearance time [227]. Based on these recent results, the World Health Organization (WHO) has not recommended systemic corticosteroids administration for COVID-19 patients outside of clinical trial studies [228]. Indeed, according to the guidelines for COVID-19 patient management released by the Center for Disease Control and Prevention (CDC), corticosteroid treatment is not recommended in confirmed COVID-19 infections except in cases with exacerbation of chronic obstructive pulmonary disease or septic shock [151].

However, recently the randomized evaluation of COVID-19 therapy (RECOVERY) trial (NCT04381936) found that dexamethasone (6 milligrams/day for 10 days) can save lives in intubated COVID-19 patients. Dexamethasone treatment reduced the 28-day mortality by one-third in intubated patients with invasive ventilation and by one-fifth in cases of patients receiving oxygen; however, it was not effective for less severe patients who did not required respiratory support [229,230]. Therefore, reflecting its benefit, the NIH COVID-19 treatment guidelines panel recommended administration of dexamethasone (6 mg/day for up to 10 days) for mechanically ventilated and oxygen supplemented COVID-19 patients. However, the possible adverse effects of secondary infections and hypoglycemia should be considered by the treating clinicians [2].

### 10.3. Nitric Oxide

Nitric Oxide (NO) is a free radical with vasodilator properties. In vascular smooth muscle cells, NO activates soluble guanylate cyclase resulting in production of cyclic guanosine monophosphate (cGMP). The latter, acting as a second messenger, activates cGMP-dependent kinase, leading to reduced cellular calcium level and vasodilation [231]. In vitro studies have reported antiviral activity of the nitric oxide donor compound S-nitroso-N-acetylpenicillamine for inhibition of SARS-Co-V replication in Vero E6 cells [232,233]. Nitric oxide inhalation has also been shown to have long term protective effect (six months after treatment) in ARDS with regard to pulmonary function [234]. Nitric oxide can inhibit both RNA and protein synthesis of SARS-CoV in vitro [232]. Moreover, nitric oxide or its derivatives can inhibit palmitoylation of the viral spike protein, affecting virus binding to its receptor ACE2 and inhibiting SARS-Co-V replication [235]. Nitric oxide inhalation has been successfully used to treat SARS patients, resulting in elevated oxygen saturation, and reduced or discontinued ventilation support [236]. There are several clinical trials currently investigating the therapeutic potential of nitric oxide therapy in patients with COVID-19 (NCT04358588, NCT04305457), as more data are required to confirm its effectiveness.

## 11. Conclusions and Future Directions

Diverse antiviral drugs with proven activity against influenza virus, SARS or MERS coronaviruses have been investigated for the treatment of COVID-19, alone or in combination with other drugs (anti-parasitic agents, anti-inflammatory drugs). Of these, remdesivir has been approved by the FDA for emergency use in adults and children hospitalized with severe, suspected or laboratory-confirmed COVID-19. Moreover, the NIH panel recommends remdesivir for treatment of COVID-19 in patients who are on mechanical ventilation or extracorporeal membrane oxygenation (ECMO) [2]. Dexamethasone treatment showed promising results in reducing the 28-day mortality in intubated patients and in cases receiving oxygen. The WHO reported that it could be lifesaving for critically ill COVID-19 patients and the NIH panel recommends using dexamethasone at proven doses in COVID-19 patients who are mechanically ventilated, as well as in patients who require supplemental oxygen but are not mechanically ventilated [2].

Early data on administration of hydroxychloroquine often in combination with azithromycin were promising, however studies were very low quality, with methodological concerns raised including small subject numbers, poor study design, the absence of appropriate controls and end points of unclear utility. Therefore, on 4 July 2020 the WHO accepted the recommendation from the Solidarity Trial’s International Steering Committee to discontinue the hydroxychloroquine arm in the trial. The fusion inhibitor umifenovir and protease inhibitor lopinavir /ritonavir were also regarded initially as likely effective candidates to treat COVID-19. However, the NIH panel now recommends against the use of chloroquine, hydroxychloroquine, Lopinavir/ritonavir or non-SARS-CoV-2-specific intravenous immune globulin (IVIG) for the treatment of COVID-19, except in a clinical trial. Furthermore, NIH does not recommend either for or against the use of COVID-19 convalescent plasma, SARS-CoV-2 immune globulin or Interleukin-6 inhibitors (e.g., sarilumab, siltuximab, tocilizumab) for the treatment of COVID-19, due to insufficient data.

The constantly changing recommendations from major international health bodies highlights the challenges of treating a novel deadly pathogen in the midst of a rapidly escalating pandemic. However, it also reinforces the importance of undertaking large, well designed clinical trials to evaluate potential treatments, instead of relying on preliminary unpublished data or the latest rumors on social media. The widespread empiric use of unproven agents such as hydroxychloroquine can lead to supply shortages for people who rely on the drug, potentially life-threatening side effects, and delays in evaluating its true clinical efficacy, along with that of other potential therapies. Many large trials are now underway to assess both novel and repurposed drugs to treat COVID-19, and their outcomes are eagerly awaited. Given the scale of the pandemic even incremental improvements can save thousands of lives, while we await the development of potent antiviral agents, targeted immunotherapies or an effective vaccine.

## Figures and Tables

**Table 1:** List of available drugs targeting SARS-CoV-2 infection and their effective dosage.

Name of Medicine	Target	Classification	Effective Dosage	Registered Clinical Trial	Reference
Remdesivir	Viral RNA polymerase	Anti-viral	IV injection 200 mg at day first, 100 mg for 9 days	NCT04257656, NCT04252664, NCT04292730, NCT04315948, NCT04321616	[237]
Lopinavir/Ritonavir	Viral protease	Anti-viral	Oral administration 400 mg lopinavir and 100 mg ritonavir twice a day for 14 days, peroral	ACTRN12620000445976, NCT02735707, ISRCTN83971151, NCT04321174, NCT04350684, ISRCTN50189673, NCT04315948, NCT04328012, NCT04276688, ISRCTN50189673, NCT04321993	[114]
Favipiravir	Viral RdRP	Anti-viral	Oral administration 1600 mg twice daily on first day and 600 mg twice a day on day 2−14.	2020-001435-27, NCT04359615, NCT04303299, NCT04402203	[120]
Ribavirin	viral RNA	Anti-viral	500 mg each time, 2 to 3 times/day in combination with IFN-α or lopinavir/ritonavir	NCT04276688, NCT04392427, ChiCTR2000029387	[238]
Arbidol	Viral RNA polymerase	Anti-viral	200 mg three times a day for a duration of 7–14 days	NCT04260594, NCT04350684, NCT04255017	[239]
Camostat	TMPRSS2	Anti-viral	600 mg (200 mg, three times)	NCT04353284	[240]
Nafamostat	TMPRSS2	Anti-viral	240 mg daily, for 5 days, peroral	NCT04418128	[138]
Chloroquine Phosphate	ACE2	Anti-parasite	500 mg (300 mg for chloroquine) each time, 2 times/day	NCT04303507, NCT04324463, NCT04353336, NCT04328493	[238]
Hydroxychloroquine	Endosome, pH elevation	Anti-parasite	200 mg, three times per day for ten days	NCT04261517, NCT04308668, NCT02735707, ISRCTN83971151, NCT04315948, NCT04321616, NCT04350684	[158,239]
IVIG	immune modulation	Immunoglobulin	400 mg/kg for a duration of five days in children	NCT04411667, NCT04261426	[241]
Ivermectin	Inhibition of nuclear transport	Anti-parasite	Oral administration 600 μg/kg) daily for 3 days	NCT04343092, NCT04392427	[242]
Tocilizumab	IL-6 receptor subunit alpha	Monoclonal antibody	400 mg intravenous or 8 mg/kg × 1–2 doses. Second dose 8–12 h after first dose if inadequate response.	NCT04335071, ChiCTR2000030894	[239]
Azitromycin	23S rRNA	Anti-microbial	500 mg on day 1 followed by 250 mg/day for the next four days (in combination with hydroxychloroquine	NCT04359316, NCT04332107, NCT04336332, NCT04329832	[158]
Corticosteroides	Binds glucocorticoid receptor and suppress inflammation	Anti-Inflammation	40 mg methylprednisolone once or twice daily	NCT04273321	[226]
Dexamethasone	Binds glucocorticoid receptor and suppress inflammation	Anti-Inflammation	6 milligrams/day for 10 days	ISRCTN50189673, NCT04381936	[243]
Nitricoxide	Activates cGMP	vasodilator	For SARS patients; Inhalation for ≥3 days (30 ppm on day 1, 20 and 10 ppm on days 2 & 3)	NCT04383002, NCT04338828, NCT04358588, NCT04305457	[236]

TMPRSS2, transmembrane protease serine 2; ACE2, angiotensin converting enzyme II, IFN-α, interferon-α.

## Abbreviations

COVID-19	Coronavirus disease 2019
SARS-CoV-2	severe acute respiratory syndrome coronavirus 2
FDA	US Food and Drug Administration
MERS-CoV	Middle East respiratory syndrome coronavirus
ARDS	acute respiratory distress syndrome
RSCU	synonymous codon usage
NSP	non-structural proteins
ORF	open reading frame
S	spike glycoprotein
E	envelope protein
M	matrix protein
N	nucleocapsid protein
RBD	receptor binding domain
ACE2	angiotensin converting enzyme II
Cryo-EM	cryo-electron microscopy
HAT	human airway trypsin-like protease
ssRNA	single stranded RNA
TMPRSS2	transmembrane protease serine 2
PLpro	papain-like proteases
3CLpro	chymotrypsin-like protease
RTC	replication–transcription complex
RdRp	RNA-dependent RNA polymerases
ER	endoplasmic reticulum
TI IF	type I interferons
IFN-α	interferon-α
Bcl-xL	B-cell lymphoma-extra large
PAMPs	pathogen associated molecular patterns
TLR	toll-like receptors
IRF3	IFN regulatory factor 3
IL	interleukin
TNF	tumor necrosis factor
RLRs	RIG-I-like receptors
CLRs	C-type lectin-like receptors
NLR	NOD-like receptor
APCs	antigen presenting cells
MHC	major histocompatibility complex
HLA	human leukocyte antigen
CTLs	cytotoxic T lymphocytes
CD	cluster of differentiation
DCs	dendritic cells
MCP-1	monocyte chemoattractant protein-1
CRS),	cytokine release syndrome
CRP	C-reactive protein
sIL-6R	IL-6 receptor
JAK-STAT3	Janus kinase-signal transducer and activator of transcription 3
Tregs	regulatory T cells
RDV	Remdesivir
EC50	half-maximal effective concentration
CC50	half-cytotoxic concentration
LPV/r	Lopinavir/Ritonavir
HIV-1	human immunodeficiency virus-1
ESICM	The European Society of Intensive Care Medicine
SCCM	The Society of Critical Care Medicine
NIH	The National Institutes of Health
FPV	Favipiravir
ARB	Arbidol
HCV	hepatitis B virus, hepatitis C virus
HA	hemagglutinin
HCoV-NL63	human coronavirus NL63
DENV	dengue virus
VEEV	Venezuelan equine encephalitis virus
IN	integrase protein
IMP	importin
mAbs	monoclonal antibodies
ERGIC	ER–Golgi intermediate compartment
IVIG	intravenous immunoglobulin
PE	plasma exchange
ICU	intensive care unit
PCR	polymerase chain reaction
qRT-PCR	quantitative reverse transcriptase PCR
WHO	World Health Organization
PD-1	programmed death-1
CDC	left for disease control and prevention
NO	nitric oxide
cGMP	cyclic guanosine monophosphate
GM-CSF	granulocyte-monocyte stimulating factor
